# Supplementary material for: Targeting Cellular Retinoic Acid Binding Protein 1 with Retinoic Acid-like Compounds to Mitigate Motor Neuron Degeneration
Source: Int J Mol Sci. 2023 Mar 4;24(5):4980. doi: 10.3390/ijms24054980 (PMC10002585; doi:10.3390/ijms24054980)
Supplement: Supplementary file 1 [file ijms-24-04980-s001.zip › ijms-2195150-supplementary.pdf]

A

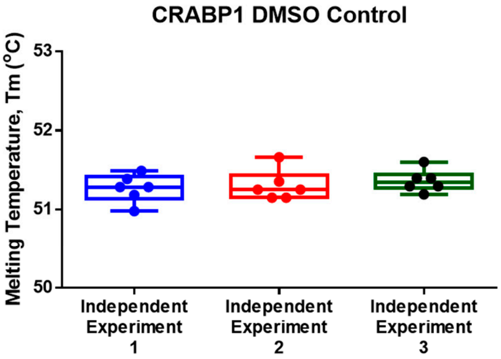

B

	DSF Exp. 1	DSF Exp. 2	DSF Exp. 3
Mean Tm (°C)	51.27	51.30	51.36
Standard Deviation	0.18	0.19	0.14

C

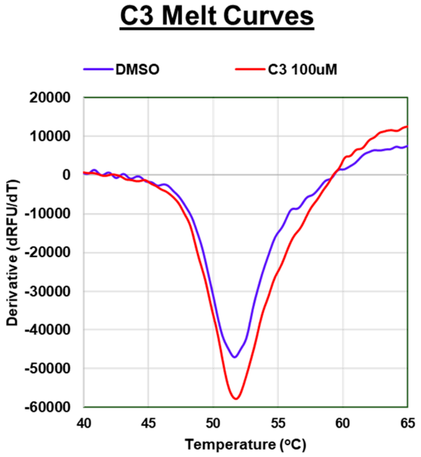

C3 Tm Shift Values		
Condition	Tm (°C)	ΔTm
DMSO	51.36	--
100uM	51.62	0.26

D

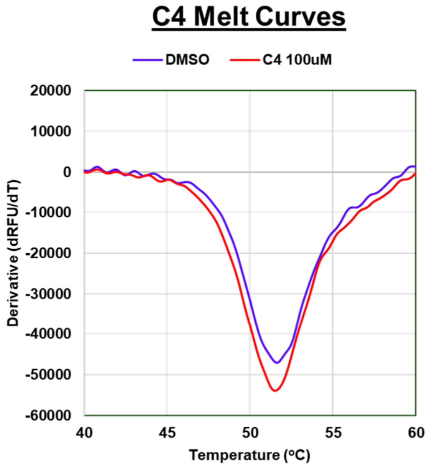

C4 Tm Shift Values		
Condition	Tm (°C)	ΔTm
DMSO	51.36	--
100uM	51.45	0.09

Supplementary Figure S1. CRABP1 DMSO controls across independent and technical replicates and C3 and C4 in CRABP1 ligand binding DSF experiments. A) Box and whisker plots of the vehicle (DMSO) controls for CRABP1 melting temperature (Tm) across 3 independent experiments. Each invididual data point represents a technical replicate. B) Mean Tm and standard deviation (SD) for each independent DMSO control. Error bars are represented as ± SD. C-D) Representative melt curve profile and Tm for C3 (100uM, ΔTm= 0.26) and C4 (100uM, ΔTm= 0.09) DSF experiments.

A

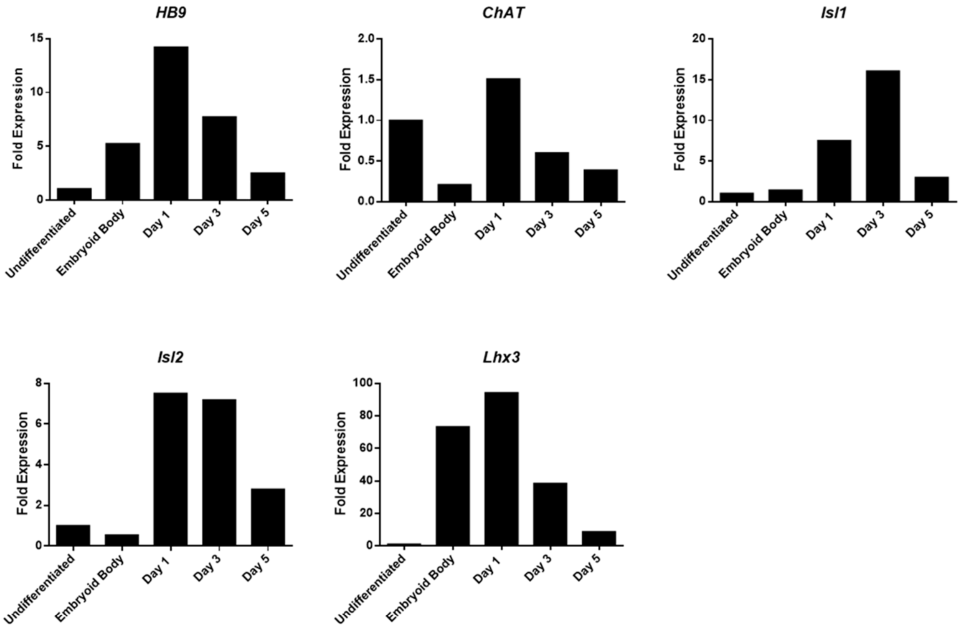

B

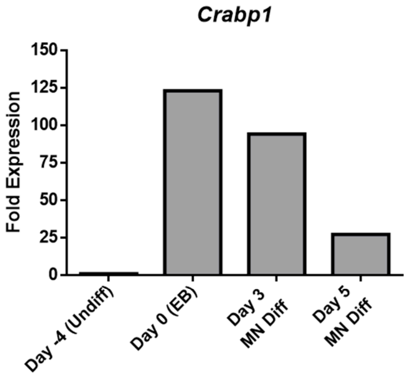

Crabp1 **Gene Expression**		
**Stage**	**Day in Vitro**	**Avg. Raw Ct**
Undifferentiated P19	-4	25.3
EB	0	17.8
Motor Neuron	3	19.1
Motor Neuron	5	20.5

C

Crabp1 **Gene Expression**		
**Stage**	**Day in Vitro**	**Avg Raw CT**
Undifferentiated P19	Day -4	24.7
EB	Day 0	18.5
Motor Neuron	Day 1	19.4
Motor Neuron	Day 3	19.5

Supplementary Figure S2. Relevant gene expression patterns of MN, spinal neuron markers, and *Crabp1* during the course of P19-MN differentiation. A) qPCR data of MN-specific markers, *HB9, ChAT, Isl1, Isl2*, and *Lhx3*, during P19-MN differentiation. B) qPCR data and raw Ct values observed for endogenous Crabp1 expression during P19-MN differentiation. C) Raw Ct values for qPCR data presented in main Figure 3D.

Supplementary Table S1. Primer sequences used in qPCR analysis

Gene Name	Primer Direction	Primer Sequence
HB9	Forward	5′-TTCCAGAACCGCCGAATGAA-3′
	Reverse	5′-CCTTCTGCTTCTCCGCCTC-3′
ChAT	Forward	5′-ACTGGGTGTCTGAGTACTGG-3′
	Reverse	5′-TTGGAAGCCATTTTGACTAT-3′
Crabp1	Forward	5′-ACCTGGAAGATGCGCAGCAGCGAG-3′
	Reverse	5′-TAAACTCCTGCATTTGCGTCCGTCC-3′
Isl2	Forward	5'-GTGCTTCGTGAGAGACGGGAAA -3'
	Reverse	5'-AGCACTCGATGTGGTACACGCT-3'
Isl1	Forward	5'-GTAGAGGTGCAAAGTTACCAGCC -3'
	Reverse	5'-TTAGAGCCTGGTCCTCCTTCTG -3'
Lhx3	Forward	5'-GAAGTTCAGGGTCGGAGGGC-3'
	Reverse	5'-TGCACACATCGGGATCTCTC-3'
